# Orthodontic bonding procedures significantly influence biofilm composition

**DOI:** 10.1186/s40510-020-00314-8

**Published:** 2020-06-01

**Authors:** Da-Mi Jeon, Jung-Sub An, Bum-Soon Lim, Sug-Joon Ahn

**Affiliations:** 1 Department of Orthodontics, School of Dentistry, Seoul National University, 101 Deahak-ro, Jongro-Gu, Seoul, 03080 Republic of Korea; 2 Department of Orthodontics, Seoul National University Dental Hospital, Jongro-Gu, Seoul, 03080 Republic of Korea; 3 Dental Research Institute and Department of Dental Biomaterials, Seoul National University Dental Hospital, Jongro-Gu, Seoul, 03080 Republic of Korea; 4 Dental Research Institute and Department of Orthodontics, Seoul National University School of Dentistry, 101 Daehakro, Jongro-Gu, Seoul, 03080 Republic of Korea

**Keywords:** Orthodontic bonding, Surface roughness, Surface wettability, Biofilm, Composition

## Abstract

**Background:**

Because changes in surface properties affect bacterial adhesion, orthodontic bonding procedures may significantly influence biofilm formation and composition around orthodontic appliances. However, most studies used a mono-species biofilm model under static conditions, which does not simulate the intraoral environment and complex interactions of oral microflora because the oral cavity is a diverse and changeable environment. In this study, a multi-species biofilm model was used under dynamic culture conditions to assess the effects of the orthodontic bonding procedure on biofilm formation and compositional changes in two main oral pathogens, *Streptococcus mutans* and *Porphyromonas gingivalis*.

**Methods:**

Four specimens were prepared with bovine incisors and bonding adhesive: untreated enamel surface (BI), enamel surface etched with 37% phosphoric acid (ET), primed enamel surface after etching (PR), and adhesive surface (AD). Surface roughness (SR), surface wettability (SW), and surface texture were evaluated. A multi-species biofilm was developed on each surface and adhesion amounts of *Streptococcus mutans*, *Porphyromonas gingivalis*, and total bacteria were analyzed at day 1 and day 4 using real-time polymerase chain reaction. After determining the differences in biofilm formation, SR, and SW between the four surfaces, relationships between bacteria levels and surface properties were analyzed.

**Results:**

The order of SR was AD < PR < BI < ET, as BI and ET showed more irregular surface texture than PR and AD. For SW, ET had the greatest value followed by PR, BI, and AD. *S. mutans* and *P. gingivalis* showed greater adhesion to BI and ET with rougher and more wettable surfaces than to AD with smoother and less wettable surfaces. The adhesion of total bacteria and *S. mutans* significantly increased over time, but the amount of *P. gingivalis* decreased. The adhesion amounts of all bacteria were positively correlated with SR and SW, irrespective of incubation time.

**Conclusions:**

Within the limitations of this study, changes in SR and SW associated with orthodontic bonding had significant effects on biofilm formation and composition of *S. mutans* and *P. gingivalis*.

## Background

Oral biofilm is a highly complex and structured microbial system, and interactions between biofilm microbes are critical to virulence [1, 2]. Microbial interactions and specific microorganisms are associated with infectious oral diseases such as dental caries and periodontal disease. In particular, a high prevalence of *Streptococcus mutans* in biofilm is associated with enamel demineralization and dental caries, and *Porphyromonas gingivalis* plays a key role in progression of gingival and periodontal inflammation by disruption of host tissue homeostasis [3, 4].

Previous study reported that orthodontic appliances can change oral microbiota with an increase in oral pathogens , such as cariogenic streptococci and periodontopathic gram-negative bacteria [4]. This may be due to the fact that patients undergoing orthodontic treatments with fixed appliances have difficulty performing proper oral hygiene, which contributes to extensive formation of oral biofilm [5]. Substantial biofilm formation and alteration of oral microbiota associated with fixed orthodontic appliances can induce enamel demineralization and gingival inflammation during orthodontic treatment; prevention of these complications is a continuous challenge faced by orthodontists [4, 6].

When bonding orthodontic appliances to the teeth, the enamel surface is subjected to many treatments including acid etching, priming, and application of adhesive to the primed surface. The purpose of these treatments is to change the tooth surface properties to increase the bond strength [7, 8]. Because surface roughness (SR) and surface wettability (SW) of biomaterials can affect biofilm formation [3, 4, 9–13], the changes in surface properties associated with the orthodontic bonding procedure may significantly influence biofilm development around orthodontic appliances.

Enamel demineralization and gingivitis are the two most common orthodontic complications associated with biofilm formation around orthodontic appliances [3, 6]. To understand these complications, some studies have analyzed the effects of orthodontic bonding on bacterial adhesion [3, 9, 12, 14]. However, most studies used a mono-species biofilm model (mainly *S. mutans*) under static conditions [9, 12, 14], which does not simulate the intraoral environment and complex interactions of oral microflora. In addition, few studies have evaluated adhesion and biofilm formation of *P. gingivalis* associated with orthodontic bonding. The purpose of this study was to analyze the effects of changes in SR and SW during orthodontic bonding procedures on biofilm formation and compositional changes in two major oral pathogens, *S. mutans* and *P. gingivalis*, using a multi-species biofilm model with dynamic culture conditions. The null hypothesis was that orthodontic bonding would have no significant effect on biofilm composition.

## Methods

### Study sample

Sound bovine incisors were extracted, cleaned, pumiced, and stored in 1% aqueous solution of chloramine-T (Junsei Chemical, Tokyo, Japan) at 4 °C. After careful preparation to a uniform size (7.5 mm diameter and 2.6 mm thickness), the bovine incisor specimens were randomly divided into three groups according to surface treatment: no surface treatment control (BI), acid-etched surface (ET), and primed surface (PR). The ET specimens were etched with 37% phosphoric acid gel (3 M, Monrovia, CA, USA) for 20 s, rinsed, and air-dried. In the PR group, Transbond XT primer (3 M) was applied to the etched surface and light-cured for 30 s using OrthoLuxLED (3 M) after acid etching. For the adhesive specimen group (AD), Transbond XT adhesive (3 M) specimens were prepared to the same size as the bovine incisor specimens using a Teflon template. The template was placed on top of a glass slide and filled with Transbond XT adhesive to flush with the top of the plate. The next slide was placed on top of the adhesive and pressed to produce a flat surface of adhesive. They were then light-cured for 20 s each from the top and bottom according to the manufacturer’s instructions. A total of 76 disk-shaped specimens (19 specimens per group) was used in this study: 72 (18 per group) for surface analyses and biofilm formation and 4 (one per group) for scanning electron microscopy (SEM) analysis.

### Surface analysis

To determine surface properties, SR and SW were measured from all 72 specimens prior to the biofilm experiment. After drying, SR of each specimen was evaluated using a confocal laser scanning microscope (LSM 5 Pascal, Carl Zeiss MicroImaging GmbH, Göttingen, Germany) to allow calculation of the arithmetic mean SR from a mean plane in the sampling area (230 × 230 × 30 μm). The measurements were performed at three random points of each disk.

SW was determined by water contact angle, as measured using a sessile drop method with distilled deionized water. Since the degree of wetting increases as contact angle decreases, the contact angle is a useful inverse measurement of SW [15]. A video camera with an image analyzer (Phoenix 300; Surface Electro Optics, Suwon, Korea) visualized the shape of the drop and determined the contact angle. The right and left contact angles of each drop were averaged. All specimens were examined by the same operator.

Surface texture of each specimen was examined using SEM. Each surface was observed under a S-4700 microscope (Hitachi, Tokyo, Japan). Representative images were collected at × 500 and × 3000 magnifications.

### Bacterial preparation

Because of their major prevalence in oral biofilm and relevance to health, a bacterial consortium of 13 species was used as previously described [16]: *Actinobacillus actinomycetemcomitans* ATCC 43718, *Actinomyces naeslundii* KCOM 1472, *Fusobacterium nucleatum* ATCC 10953, *Lactobacillus rhamnosus* ATCC 7469, *Neisseria subflava* ATCC 49275, *P. gingivalis* KCOM 2797, *Prevotella nigrescens* ATCC 33563, *S. mutans* ATCC 700610, *Streptococcus oralis* ATCC 9811, *Streptococcus salivarius* CCUG 50207, *Streptococcus sanguinis* CCUG 17826, *Streptococcus sobrinus* ATCC 27607, and *Veillonella dispar* KCOM 1864. Since these species have different optimal growth environments, they were individually grown to mid-exponential phase according to growth nature (Table 1).

**Table 1:** Growth conditions of each bacterial species for the multi-species biofilm model

Bacterial species	Growth condition
*Streptococcus mutans*, *Streptococcus sobrinus, Streptococcus sanguinis*, *Streptococcus salivarius, Streptococcus oralis*, *Actinomyces naeslundii, Lactobacillus rhamnosus*, *Veillonella dispar*, *Neisseria subflava*	Brain heart infusion medium at 37 °C with 5% CO_2_
*Fusobacterium nucleatum*, *Prevotella nigrescens*, *Porphyromonas gingivalis*	Anaerobic condition with tryptic soy agar medium supplemented with 10 μg/mL vitamin K, 5 μg/mL hemin, and 5% sheep blood at 37 °C for 7 days Subcultured in BHI medium with 10 μg/mL vitamin K and 5 μm/mL hemin and grown to mid-exponential phase anaerobically at 37 °C
*Actinobacillus actinomycetemcomitans*	Brain heart infusion medium at 37 °C in an anaerobic condition

### Multi-species biofilm formation

We cultivated the multi-species biofilm using a CDC biofilm reactor (BioSurface Technologies, Bozeman, MT, USA) with a modified basal mucin medium to provide nutrients and simulate saliva [17]. The medium contained 2.5 g/L porcine gastric mucin, 2 g/L proteose peptone, 1 g/L yeast extract, 1 g/L trypticase peptone, 2.5 g/L KCL, 0.1 g/L cysteine hydrochloride, 0.001 g/L hemin, 10 mM urea, and 10 mM glucose. The reactor has a lid supporting eight rods that each held three individual specimens. Three specimens were randomly selected from each of the four groups and inserted into each rod using a Teflon template to expose only the front surface to the culture medium. The equipment, the rods with specimens, and the basal medium mucin were then sterilized. After 3.5 mL of prepared mixed cell culture (1% of the reactor volume) was injected into the biofilm reactor, modified basal mucin medium was continuously pumped into and flowed out of the reactor at a rate of 100 mL/h. The reactor was set on a hot stir plate at 37 °C with a rotational speed of 60 rpm as previously described [18].

### Quantitative analysis of bacteria

The 12 specimens were removed from the biofilm reactor at day 1 (T1) and day 4 (T2). The specimens were transferred into round tubes and washed twice with 1.0 mL phosphate-buffered saline (PBS; pH = 7.4) for removing unbound bacteria. Through sonication with three 30-s pulses and 30-s intermittent ice cooling procedures, the biofilm was detached from the specimen surface. The bacterial suspension was then centrifuged at 13,000 rpm for 10 min and washed with 1.0 mL PBS.

Bacterial chromosomal DNA was extracted using a CellEase Bacteria II Genomic DNA Extraction Kit (Biocosm, Osaka, Japan). A NanoVue spectrophotometer (General Electric Healthcare Life Sciences, Pittsburgh, PA, USA) was then used to estimate the quality of the isolated DNA. For quantifying *S. mutans*, *P. gingivalis*, and total bacteria in biofilm using real-time polymerase chain reaction (PCR), PCR primers were commercially synthesized from Bioneer (Daejeon, Korea) to amplify the target DNA. The specific primers for *S. mutans* were designed from the *gtfB* and *gtfU* genes, and the primers of *P. gingivalis* were based on the 16S rRNA gene as previously described [3]. A conserved sequence in the 16S rRNA was selected for quantifying total bacteria as previously described [3].

To obtain the standard curve for DNA quantification, DNA was isolated from *S. mutans* ATCC 700610 and *P. gingivalis* KCOM 2797 and amplified. The amplified products were purified from agarose gels by a QIAquick Gel Extraction Kit (Qiagen, Düsseldorf, Germany), and DNA concentration was determined from absorbance at 260 nm. A 10-fold serial dilution ranging from 10 to 10^7^ copies was performed to create DNA standard curves.

Real-time PCR was performed with the Bio-Rad iQ5 system (Bio-Rad, Hercules, CA, USA). The reaction mixtures contained 2 μL purified DNA from the specimens, 100 pM primer, and 10 μL 2x iQ SYBR Green Supermix (Bio-Rad). Distilled water was added to a final volume of 20 μL. Thermal cycling conditions for quantifying target bacteria are presented in Table 2. Bio-Rad iQ5 Optical System Software was used to analyze all data. The entire quantifying procedure was performed in duplicate and individually repeated five times.

**Table 2:** Polymerase chain reaction conditions with respect to bacterial species

Bacterial primer	Cycling condition
Primers for *Streptococcus mutans*, Universal primers	Initial denaturation for 30 s at 94 °C Forty cycles of denaturation for 20 s at 95 °C Annealing for 45 s at 60 °C Extension for 10 s at 60 °C
Primers for *Porphyromonas gingivalis*	Initial denaturation for 1 min at 95 °C Forty cycles of denaturation for 5 s at 95 °C Annealing for 15 s at 61 °C Extension for 33 s at 72 °C Final extension for 10 min at 72 °C

### Statistical analysis

The Kruskal-Wallis test was used to determine differences in SR and water contact angle according to surface treatment. Two-way analysis of variance using the Bonferroni correction was used to determine the differences in the levels of bacteria with respect to incubation time and surface type. Spearman rank correlation coefficient test was used to examine the associations between surface properties and bacterial levels at each time point. All values were considered significant at *P* < 0.05.

## Results

**Fig. 1:**
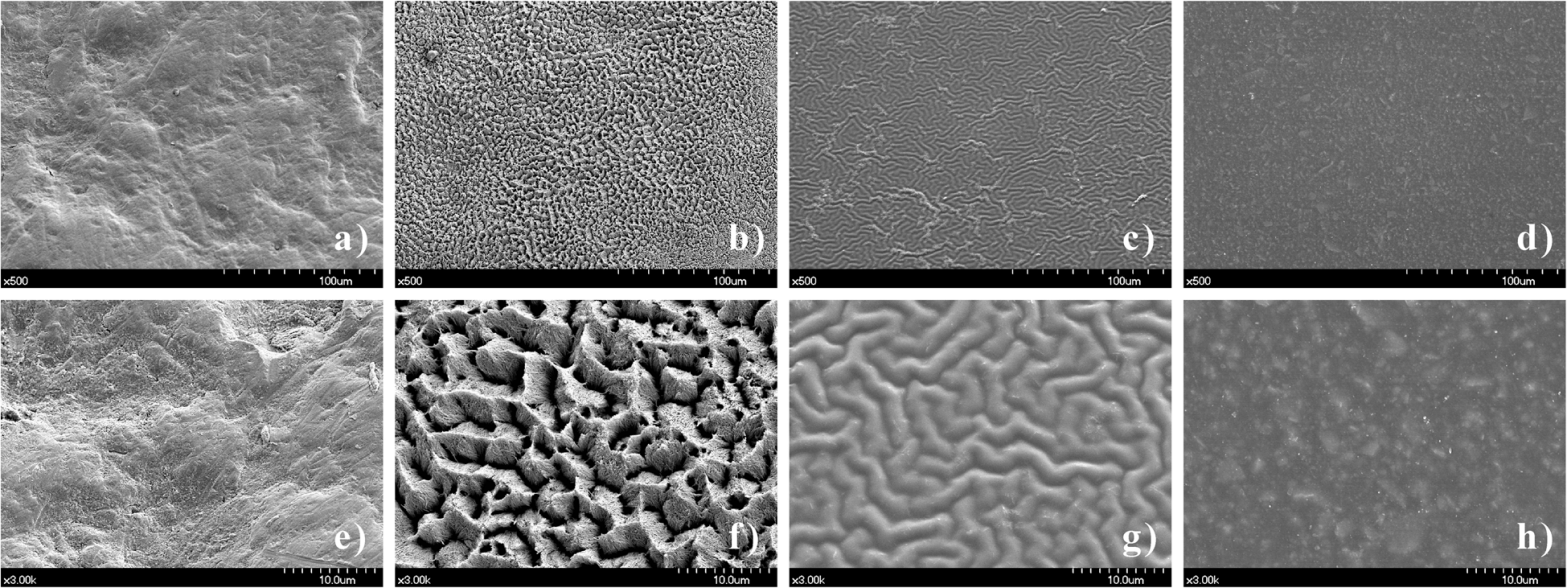
Scanning electron microscopy images with respect to surface type. **a** Untreated bovine incisor at × 500 magnification; **b** etched bovine incisor at × 500 magnification; **c** primed bovine incisor at × 500 magnification; **d** Transbond XT adhesive at × 500 magnification; **e** untreated bovine incisor at × 3000 magnification; **f** etched bovine incisor at × 3000 magnification; **g** primed bovine incisor at × 3000 magnification; **h** Transbond XT adhesive at × 3000 magnification

There was a significant difference in SR among the surface types (Table 3). ET had the roughest surface, while AD had the smoothest surface. Multiple comparisons showed that the order of SR was AD < PR < BI < ET (*P* < 0.05), consistent with results from SEM images. BI and ET showed rougher surface textures than PR and AD (Fig. 1). BI had an irregular and uneven appearance due to grooves, ridges, and microfissures (Fig. 1a, e). Because of dissolution of the prism core, ET showed many microporosities throughout the surface (Fig. 1b, f). PR showed a uniformly wrinkled surface and rougher texture (Fig. 1c, g) than AD with minor flaws (Fig. 1d, h).

**Table 3:** Surface roughness and water contact angle with respect to surface type

	Bovine incisor (BI)	Etching (ET)	Primer (PR)	Transbond XT (AD)	Multiple Comparisons
Surface roughness (μm)	1.61 ± 0.22	3.50 ± 0.30	0.32 ± 0.04	0.11 ± 0.00	AD < PR < BI < ET
Water contact angle (degree)	56.61 ± 2.50	30.08 ± 2.94	46.59 ± 1.51	72.47 ± 1.62	ET < PR < BI < AD

The Kruskal-Wallis test was used to determine differences among the four groups and multiple comparisons were performed using the Mann-Whitney tests with the Bonferroni correction at a significant level of *α* < 0.05.

Significant differences in water contact angle were found among the surface types, but the order of water contact angle tended to be opposite to that of SR, at ET < PR < BI < AD (*P* < 0.05) (Table 3). Because the contact angle is a useful inverse measurement of SW [15], the order of SW can be interpreted as AD < BI < PR < ET.

Table 4 exhibits the differences in bacterial adhesion according to surface type and incubation time. The surface type significantly influenced the adhesion of target bacteria. In all target bacteria, BI and ET demonstrated higher bacterial adhesion than AD, but there was no significant difference in adhesion level between BI and ET (AD < BI = ET). Bacterial adhesion to PR varied among bacterial species. There was no significant difference in adhesion amount of *S. mutans* between PR and the other three surfaces. Adhesion amount of *P. gingivalis* to PR was lower than that to ET, but there were no significant differences in adhesion amount of *P. gingivalis* among BI, PR, and AD. Total bacteria exhibited lower adhesion to PR than to BI and ET, but no significant difference in adhesion was found between PR and AD.

**Table 4:** Time-related differences in the levels of bacteria with respect to surface type

	Day 1 (T1)	Day 4 (T2)	Significance†		*P* value for interaction
			Treatment	Time	
*Streptococcus mutans* (Log_10_/cm^2^)					
BI^a^	4.29 ± 0.22	5.22 ± 0.53	AD < BI = ET	T1< T2	0.388
ET^b^	4.47 ± 0.36	5.31 ± 0.39			
PR^c^	4.05 ± 0.44	5.01 ± 0.27			
AD^d^	3.96 ± 0.37	4.49 ± 0.59			
*Porphyromonas gingivalis* (Log_10_/cm^2^)					
BI^a^	3.23 ± 0.31	2.82 ± 0.30	AD < BI = ET PR < ET	T1 > T2	0.905
ET^b^	3.48 ± 0.40	3.14 ± 0.44			
PR^c^	2.80 ± 0.28	2.52 ± 0.53			
AD^d^	2.57 ± 0.17	2.36 ± 0.63			
Total bacteria (Log_10_/cm^2^)					
BI^a^	7.06 ± 0.34	7.99 ± 0.24	AD = PR < BI = ET	T1 < T2	0.104
ET^b^	7.25 ± 0.36	8.11 ± 0.33			
PR^c^	6.90 ± 0.48	7.70 ± 0.24			
AD^d^	6.87 ± 0.26	7.34 ± 0.42			

^†^Two-way ANOVA was used to determine significant differences between the two time points using the Bonferroni correction at a significant level of *α* < 0.05

^a^Untreated bovine incisor

^b^Etched bovine incisor

^c^Primed bovine incisor

^d^Transbond XT adhesive

There were also significant differences in bacterial adhesion between T1 and T2 (Table 4). The adhesion amounts of total bacteria and *S. mutans* increased (T1 < T2, *P* < 0.05), but that of *P. gingivalis* decreased with increased incubation time (T1 > T2, *P* < 0.05).

The Spearman rank correlation test demonstrated that the adhesion level of all target bacteria was significantly associated with both SR and water contact angle at each time point (Table 5). Bacterial adhesion was positively correlated with SR, but negatively correlated with water contact angle, irrespective of bacterial species and incubation time. Considering that water contact angle is an inverse measurement of SW [15], these results indicate that both SR and SW are positively correlated with bacterial adhesion.

**Table 5:** Spearman rank correlation coefficients for surface properties and bacterial levels

Day	Bacteria	Surface roughness (*n* = 36)	Water contact angle (*n* = 36)
1	*Streptococcus mutans*	0.590***	− 0.456**
	*Porphyromonas gingivalis*	0.863***	− 0.635***
	Total bacteria	0.599***	− 0.566***
4	*Streptococcus mutans*	0.458**	− 0.351**
	*Porphyromonas gingivalis*	0.465**	− 0.373**
	Total bacteria	0.844***	− 0.573***

**P < 0.01; ***P < 0.001

## Discussion

A common orthodontic bonding procedure includes etching the tooth surface, priming the tooth surface, applying a bracket with a bonding adhesive to the tooth surface, and curing the adhesive between the tooth and bracket. The etched surface provides increased surface area and hydrophilic properties; priming protects the etched enamel surface and enhances the bond with the adhesive [7]; and the bonding adhesive provides adequate physical strength between the bracket base and etched and primed enamel surface, resists displacement by oral function, and transfers requisite orthodontic force to the tooth [8]. Previous in vitro studies have evaluated the relationship between orthodontic bonding and biofilm formation, which is important to prevent common orthodontic complications, such as enamel demineralization and gingival inflammation [3, 5, 6, 9, 12, 14]. However, most studies only used a single bacterial species, mainly *S. mutans* [9, 12, 14]. The single-species method cannot represent interactions of microorganisms associated with oral biofilms. In this study, a multi-species biofilm model was used under dynamic culture conditions to assess the effects of the orthodontic bonding procedure on biofilm formation and compositional changes in two main oral pathogens, *S. mutans* and *P. gingivalis*.

Bovine teeth were used to examine the effects of surface properties on biofilm formation in this study because they are the most widely used alternative for human teeth in dental research. They are easy to obtain in good condition and have a relatively large flat surface. Although the physicochemical characteristics of bovine teeth are not identical to those of human teeth [19], many studies have reported that there are no significant differences in micro-morphology, physical properties, and chemical composition [20, 21]. In addition, there is no significant difference in biofilm composition between human and bovine teeth [22].

SR and SW are two main surface properties that influence bacterial adhesion and biofilm formation [3, 4, 9–13]. A rough surface provides a favorable environment for bacterial adhesion and biofilm maturation, because a rough surface plays a protective role against shear force and increases the area available for biofilm formation [11, 12]. On the other hand, higher SW facilitates biofilm formation on dental materials [10, 13] due to its relation to surface free energy and hydrophilicity [23].

SW is measured by contact angle, which is formed when a droplet of a liquid is placed on a surface [15]. Water is a common liquid to use for measurement of the contact angle because it has no chemical reaction with the underlying surface [24]. We measured the water contact angle of all the specimens to determine the SW prior to starting biofilm experiments, because other probe liquids with different hydrophobicity may affect the surface properties of the underlying material, react with primer or adhesive components, and influence biofilm experiments.

This study demonstrated that surface treatment during orthodontic bonding significantly influences SR and water contact angle. There were significant differences in SR among the surface types (Table 3). The order of SR was AD < PR < BI < ET, which is partly consistent with the results of a previous study showing that etched hydroxyapatite surface is rougher and adhesive surface is smoother than those of other surfaces [12]. Higher SRs of BI and ET than PR and AD might be due to the presence of grooves and ridges on bovine enamel and increased surface irregularities by acid etching [25], respectively (Fig. 1). Although the wrinkled surface of PR showed a smoother texture (Fig. 1c, g) than BI and ET, wrinkle structures may cause PR to be more irregular than AD, resulting in minor flaws (Fig. 1d, h).

There were also significant differences in water contact angle among the four surface types (Table 3). AD exhibited the greatest value followed by BI, PR, and ET (ET < PR < BI < AD). Because of the inverse relationship between water contact angle and SW [15], the order of SW may be AD < BI < PR < ET. These findings indicate that both SR and SW have the highest value in ET and the lowest value in AD.

This study demonstrated higher adhesion of *S. mutans* to BI and ET than to AD (Table 4), which could be explained by the higher SR and SW for BI and ET than for AD (Table 3). Various bacteria are involved in biofilm formation in the oral cavity, which begins with early colonizers, including streptococci and *Actinomyces* spp., followed by middle-colonizing *Porphyromonas* spp. and *Fusobacteria* spp., and late-colonizing Gram-negative anaerobes [1, 2]. Because *S. mutans* initially adheres to the underlying surface as an early colonizer, adhesion of *S. mutans* may be more significantly affected by surface properties. Previous microscopic examination of biofilms revealed that bacterial adhesion to the enamel surface starts from surface irregularities, such as grooves, perikymata, and cracks [25] (Fig. 1), because rough surfaces can act as a buffer against shear forces, which ease the change from reversible to irreversible bacterial attachment and increase the area available for initial bacterial adhesion. In addition, hydrophilic and wettable surfaces are known to collect more plaque by attracting specific bacteria [26, 27]. Since hydrophilic bacteria preferentially adhere to a hydrophilic surface [26], hydrophilic oral bacteria, such as *S. mutans*, easily adhere to the hydrophilic and wettable surface [27]. In this regard, the rougher and wetter surfaces of BI and ET may provide more a favorable surface for adhesion of *S. mutans* than AD.

This study showed that *P. gingivalis* and total bacteria also showed greater adhesion to BI and ET than to AD (Table 4). After colonization by early colonizers, a combination of bacterial proliferation and recruitment leads to a bacterial mass increase during biofilm maturation [2]. Therefore, successful adhesion of early colonizers such as *S. mutans* leads to sequential co-adhesion and proliferation of middle and late colonizers and results in increase and maturation of the biofilm, which may explain the similar adhesion tendency of *P. gingivalis* and total bacteria to that of *S. mutans*. This hypothesis is supported by the findings of this study demonstrating that SR was positively correlated and water contact angle was negatively correlated with adhesion of *P. gingivalis* and total bacteria as well as *S. mutans* (Table 5). Several studies have also demonstrated that SR has a positive correlation with bacterial adhesion and biofilm formation [3, 11, 12] and the significant effects of SW on biofilm formation are widely accepted [10, 13]. All these findings suggest that changes in SR and SW during orthodontic bonding procedures may significantly affect bacterial adhesion and biofilm composition.

Biofilm formation can be influenced not only by changes in SR and SW but also by other surface factors during orthodontic bonding procedures. Bacterial adhesion to ET was expected to be higher than to BI, because of its rougher and wetter properties. However, there was no significant difference in adhesion of any bacteria between BI and ET (Table 4). Cytotoxicity of the phosphoric acid used for acid etching may influence bacterial adhesion. A previous study demonstrated that 37% phosphoric acid has antimicrobial activity by increasing the concentration of hydrogen ions in the microorganism [28]. During the experiment, the remaining phosphoric acid on the irregular surface of the bovine tooth may have influenced the bacterial viability. Although the rougher and wetter surface caused by acid etching could be favorable for bacterial adhesion, the cytotoxic action of phosphoric acid may offset the surface properties. In addition, a previous study reported that an SR over a certain level (over 0.35 μm) might not significantly influence biofilm formation [29]. Although ET was rougher than BI, BI may be rough enough (average 1.61 μm of SR, Table 3) to demonstrate no difference in bacterial adhesion.

Bacterial adhesion to PR was different than that to other surfaces. Because PR was rougher and more wettable than AD, but smoother and less wettable than ET (Table 3), bacterial adhesion to PR was expected to be lower than that to ET and higher than that to AD. However, adhesion of the two oral pathogens and total bacteria to PR was not significantly different from that to ET or AD. This result may be due to chemical properties of the primer. The primer is present in a chemically unstable state in the oral environment because of its lower degree of conversion [30]. In particular, bisphenol A-glycidyl methacrylate (bis-GMA), one of the main components of Transbond XT primer, has two opposing characteristics that influence bacterial adhesion and biofilm formation. One is to facilitate biofilm formation of *S. mutans* by increased adhesion capacity, enhanced glucan synthesis, and promotion of sugar transport activity [31]. The other is a toxic effect on oral bacteria, such as inhibiting bacterial growth and decreasing cell viability [31]. The leachable components of the primer with these opposing characteristics may differently influence bacterial adhesion to PR.

This study showed that adhesion of *S. mutans* and total bacteria significantly increased with extended incubation time (T1 < T2, Table 4). Since streptococci are facultative anaerobes, they can successfully adhere to the surface and continue to proliferate well in our aerobic culture condition, which led to subsequent maturation of biofilm and eventually resulted in an increase in total bacteria. In contrast to *S. mutans* and total bacteria, the amount of *P. gingivalis* significantly decreased from T1 to T2 (T1 > T2). *P. gingivalis* is a late colonizer and obligate anaerobe. *P. gingivalis* is sensitive to an oxidative aerobic environment, possibly hindering its growth. These results are consistent with a previous study that examined biofilm formation on orthodontic adhesive under similar culture conditions to our study [3].

This in vitro study showed the lowest bacterial adhesion to AD. In particular, the two main oral pathogens showed less adhesion to AD than to BI and ET. Considering that plaque accumulation and enamel demineralization mainly occur at the interface between tooth and adhesive in clinical practice [32], these findings indicate that when acid etching is wider than intended, covering the etched surface with adhesive may be helpful to reduce biofilm formation around orthodontic appliances. However, it is difficult to maintain a smooth adhesive surface and the remaining adhesive remnant around orthodontic appliances may be difficult to clean properly in the clinical situation. Therefore, clinicians should uniformly apply adhesive, carefully remove adhesive remnants, and perform periodic cleaning around orthodontic appliances to avoid enamel demineralization.

The present study has some limitations. This study showed that there was no significant difference in bacterial adhesion between BI and ET, even though ET had a rougher and more wettable surface than BI. However, the effects of acid etching on bacterial adhesion may be different between human and bovine teeth, because bovine teeth are more irregular and undulating than human teeth [19]. In addition, the multi-species biofilm model used in this study does not simulate the actual oral environment. Further study using an in situ model is needed to evaluate the effects of orthodontic bonding procedures on biofilm formation and to find approaches to minimize the risk of pathologic side effects in orthodontic patients.

## Conclusions

This study demonstrated that surface treatment during orthodontic bonding significantly influences SR and SW. Acid etching significantly increased SR and SW, while application of adhesive significantly decreased SR and SW. The changes in SR and SW were significantly associated with biofilm formation and composition. In particular, the two main oral pathogens, *S. mutans* and *P. gingivalis*, showed greater adhesion to BI and ET with rougher and more wettable surfaces than to AD with smoother and less wettable surfaces. This in vitro study suggests that changes in surface properties during the orthodontic bonding procedure may be significantly associated with biofilm formation and composition of *S. mutans* and *P. gingivalis*.

## Data Availability

The datasets used and/or analyzed during the current study are available from the corresponding author on reasonable request.

## Abbreviations

BI: Untreated bovine enamel surface
ET: Bovine enamel surface etched with 37% phosphoric acid
PR: Primed enamel surface
AD: Adhesive surface
SW: Surface wettability
SR: Surface roughness
